# Shock Induced Endotheliopathy and High Trauma Mortality—Fight-or-Flight Response Revisited

**DOI:** 10.3390/ijms27167210

**Published:** 2026-08-12

**Authors:** Nathan Weinstein, John B. Holcomb, Pär I. Johansson

**Affiliations:** 1 CAG Center for Endotheliomics, Copenhagen University Hospital, Rigshospitalet, Blegdamsvej 9, 2100 Copenhagen, Denmark; nathan.weinstein@c3.unam.mx; 2 Department of Surgery, University of Alabama at Birmingham, 1808 7th Avenue South, Birmingham, AL 35233, USA; jbholcomb@uabmc.edu; 3 Department of Clinical Immunology, Copenhagen University Hospital, Rigshospitalet, Blegdamsvej 9, 2100 Copenhagen, Denmark

**Keywords:** traumahemorrhage, sympathetic activation, shock induced endotheliopathy, multiorgan failure

## Abstract

Trauma with hemorrhagic shock causes about 2.3 million deaths yearly worldwide. Improved hemostatic management has shifted the relative distribution of mortality, leaving multiorgan failure (MOF) as a leading cause of death. Clinical observations suggest that the evolutionarily ancient and well-conserved sympathetic system and the microvascular endothelium are involved, and the experimental evidence is reviewed here. The analyzed clinical studies, human endothelial cell (EC) culture, and animal model-based experiments delineate how excess catecholamine levels increase endothelial cell reactive oxygen species production, causing glycocalyx damage and thrombomodulin cleavage. This leads to a prothrombotic EC surface, resulting in coagulation activation and thrombus formation that leaves tissues prone to hypoxia. Excess catecholamines also increase endothelial barrier permeability, leading to fluid extravasation, elevated tissue pressure, and hypoxia. The reviewed experimental data support, but do not yet prove, dysregulated sympathetic activation’s critical contribution to the development of shock-induced endotheliopathy prone to tissue hypoxia and, ultimately, death from coagulopathy, loss of immune competence, and MOF, as observed clinically in shocked trauma patients. Due to the physiological differences between humans and model organisms, and EC culture growth conditions, some molecular mechanisms require further investigation through clinical studies and targeted experiments.

## 1. Introduction

**Figure 1:**
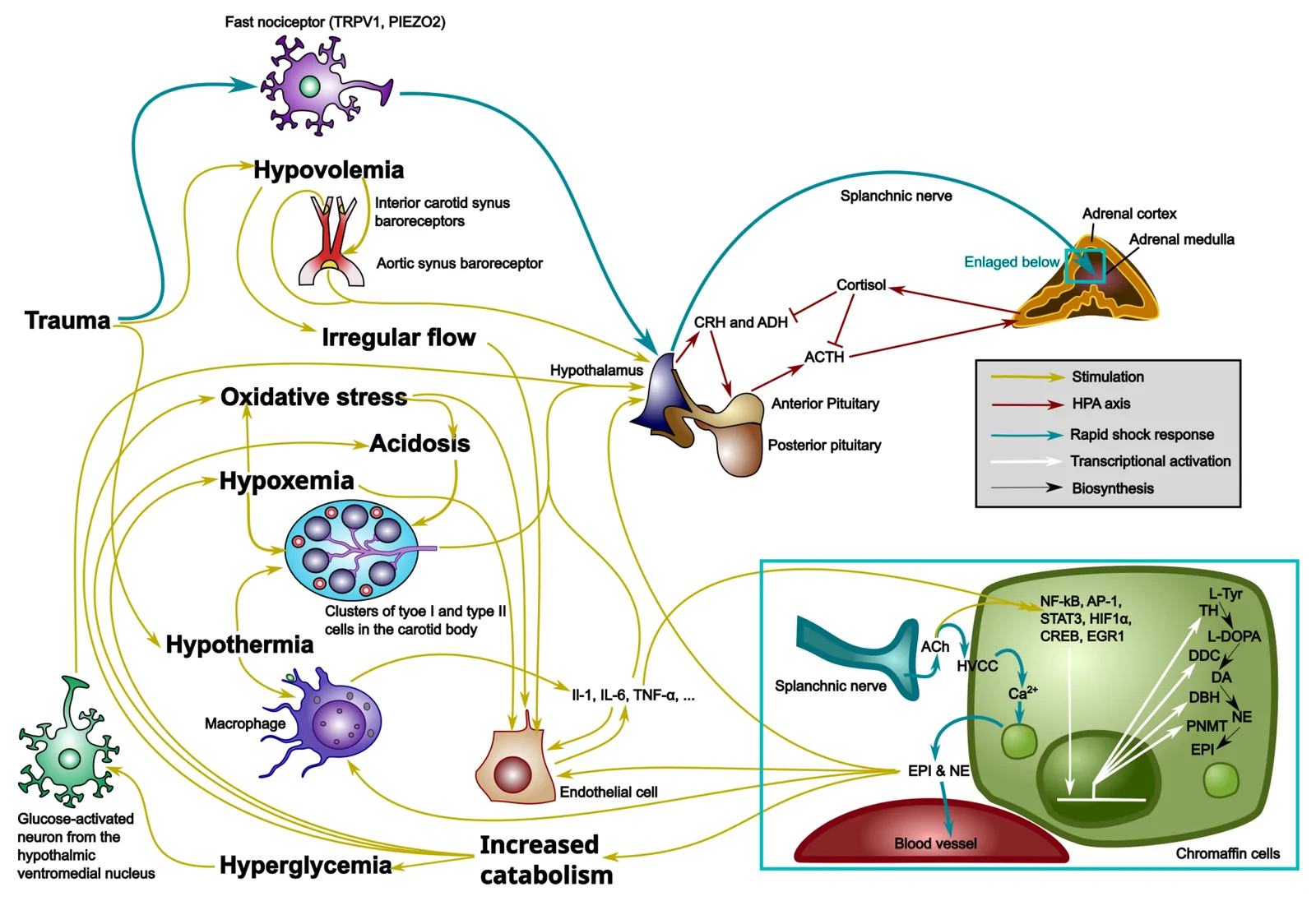
Response to trauma:Immediately after tissue injury, nociception activates sympathetic nervous system signaling through the splanchnic nerve and stimulates Chromaffin cells in the adrenal medulla, causing them to release norepinephrine (NE) and epinephrine (EPI). Later, circulatory shock-induced hypovolemia, hyperglycemia, hypoxemia, hypercapnia, hypothermia, acidosis, and cytokine release continue to activate the shock response in the hypothalamus, prolonging the release of NE and EPI, which cause endothelial dysfunction and immunosuppression. The hypothalamus also releases corticotropin-releasing hormone (CRH) and antidiuretic hormone (ADH), which cause the anterior pituitary gland to secrete adrenocorticotropic hormone (ACTH), which in turn causes the adrenal cortex to produce cortisol. Additionally, the cytokines, acetylcholine (ACh), and cortisol augment the production of EPI and NE in chromaffin cells. Endothelial cells and leukocyte populations can synthesize and release catecholamines, which in turn modulate their local response to tissue trauma.

Trauma with hemorrhagic shock contributes to nearly half of the annual 4.6 million injury deaths worldwide [1]. The introduction of hemostatic resuscitation and damage control surgery 15 years ago has significantly reduced exsanguination; however, this was followed by a concomitant increase in multiorgan failure (MOF), and the mortality rate of severely shocked patients has remained at approximately 50% [2,3,4,5]. The pathophysiology of both trauma-induced coagulopathy (TIC) and MOF remains elusive, although there is a general consensus that dysregulation of the inflammatory, immune, and hemostatic systems is critically involved in the latter, and this has recently been well reviewed elsewhere [6,7,8,9,10,11,12,13]. Briefly, traumatic shock increases inflammatory cytokine production and simultaneously reduces resistance to infection [6]. This immune response causes several extracellular factors to drive plasma membrane damage across multiple organs concurrently. These primary inducers include elevated extracellular phospholipases, reactive oxygen and nitrogen species, pore-forming proteins (PFPs), and disrupted osmotic homeostasis [11]. Unfortunately, pharmacological interventions targeting these systems in shocked trauma patients as well as sepsis patients have yielded disappointing results, suggesting that other pathophysiological mechanisms responsible for the high mortality may also be involved [2,3,4,5]. In alignment with Cannon’s landmark work a century ago introducing the sympathoadrenal system as a functional whole, mounting the “fight-or-flight” response as a strategy to cope with shock, including traumatic haemorrhage [14,15,16], observational studies in cohorts of trauma patients with hemorrhagic shock, in the early 2010’s emerged that reported independent associations between excess sympathetic activation with epinephrine (EPI) release, endothelial glycocalyx shedding, and 30-day mortality [17,18,19]. This pointed towards the involvement of the sympathetic nervous system (SNS) and the endothelium in the pathology contributing to the high mortality observed [20]. Immediately after traumatic tissue injury occurs, mechanical forces reach nociceptor cells, initiating an action potential that travels to the brain through fast, myelinated, type A high threshold mechanoreceptors, resulting in the activation of the SNS [21,22,23]. Subsequently, SNS signaling stimulates the splanchnic nerve that connects the spinal cord to the adrenal glands, causing chromaffin cells in the adrenal medulla to secrete previously stored EPI and NE into the systemic circulation [24,25] (Figure 1). SNS activation also induces the rapid release of NE stored within the sympathetic neurons that innervate several organs, including the thymus, spleen, lymph nodes, bone marrow, heart, blood vessels, bronchi, and GI [26,27,28]. The ensuing hypovolemia [29,30], hypoxemia [31], oxidative stress [32], hypercapnia, acidosis, hypothermia [33,34], hyperglycemia [35,36], and excess hypothalamic-pituitary-adrenal activity [37,38], all frequently observed in severely injured trauma patients with shock, further stimulate the SNS, resulting in persistent and elevated catecholamine release (Figure 1). The SNS, with the “fight or flight” system, goes back to the jawless vertebrates, like sea lampreys, and its core development pathways are conserved across all vertebrates as a strategy to cope with shock [39]. The findings above suggest that those trauma patients who mount an excess activation of the SNS response, including the release of excess catecholamine levels to shock, may suffer harm and that this involves an interplay with the endothelium.

**Figure 2:**
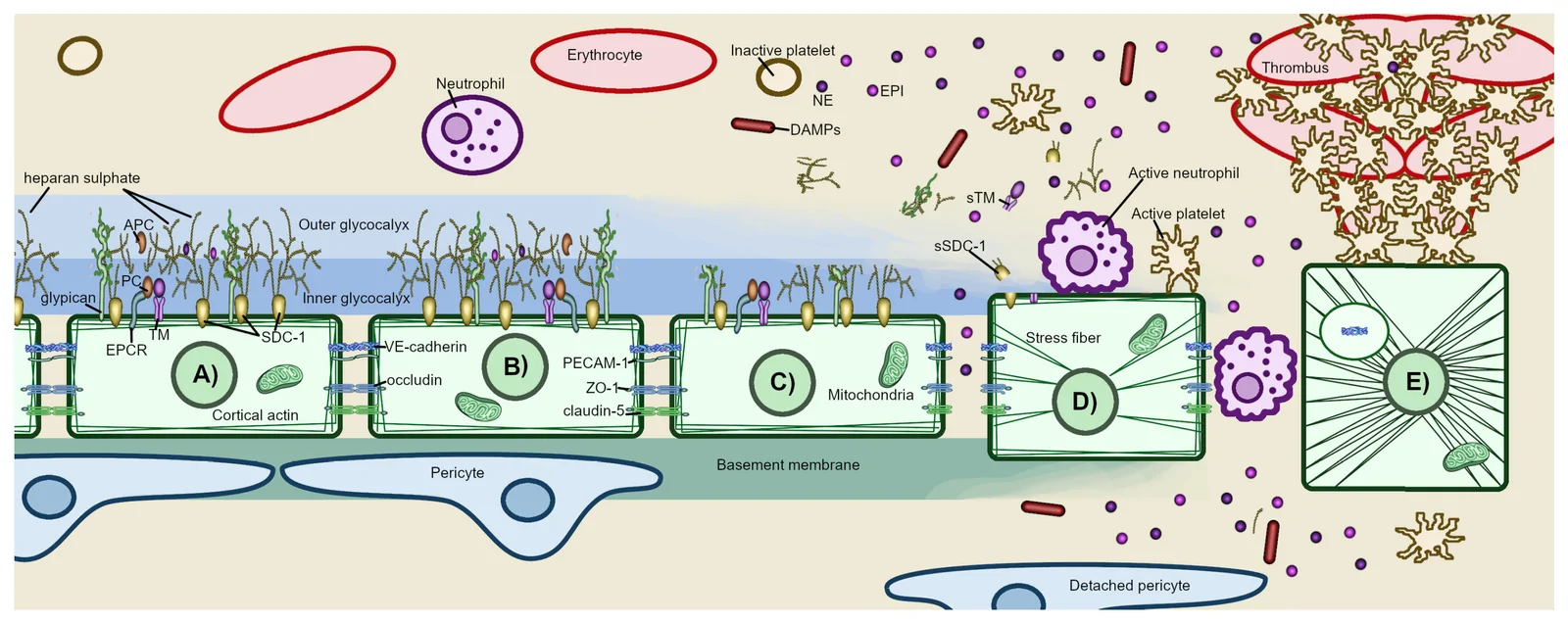
The endothelium affected by trauma: (**A**) A healthy quiescent endothelial cell (EC) where the luminal side of the membrane is covered with glycocalyx, which hinders coagulation initiation. (**B**) Healthy ECs are interconnected by stable adherence and tight junctions. (**C**) An EC showing catecholamine-induced outer glycocalyx damage. (**D**) A contracting EC with detached adherence and tight junctions. Note the inner glycocalyx damage that allows active neutrophils and platelets to adhere to the luminal EC membrane. (**E**) Endothelial barrier failure, neutrophil diapedesis, thrombus formation, and vascular endothelial cadherin (VE-cadherin) internalization.

The specialized endothelial cell (EC) lining evolved in ancient vertebrates some 540–510 million years ago to optimize flow dynamics and barrier function, and to localize immune and coagulation functions [40,41,42]. It is one of the largest “organs” in the body, weighing up to 1 kg with a surface area exceeding 1000 m^2^ [14]. ECs are essential for the regulation of vasomotor tone, blood cell extravasation, blood fluidity, vascular permeability, and angiogenesis, as well as for modulating both innate and adaptive immunity, and, pivotally, for the delivery of oxygen and nutrients throughout the body [40,43,44]. Due to its unique location and pivotal importance for vascular homeostasis, it is recognized that the endothelium is involved in most, if not all, disease states, including those induced by traumatic shock, either as a primary determinant of pathophysiology or as a victim of collateral damage [41]. As a result of more than 500 million years of co-evolvement between the SNS and the endothelial cell lining the microvascular ECs encompass all nine subtypes of adrenergic receptors [45]. Building on this and on reports firstly from patients suffering from traumatic shock, Shock Induced Endotheliopathy (SHINE) was proposed by us in 2017, as a unifying pathophysiological mechanism contributing to the high mortality observed in these patients [46]. SHINE develops secondary to the trauma -and hemorrhagic shock-induced activation of the SNS, with the release of excess levels of catecholamines (e.g., dopamine, EPI, and NE) into the bloodstream as well as NE released directly at vital tissue beds, including the microvascular endothelium [47]. The catecholamines inflict dose-dependent downstream effects on the microvascular ECs (Figure 2C), leading to the three main types of endotheliopathies, which are glycocalyx shedding, cleavage of TM into its soluble form (sTM) with protein C system impairment (Figure 2C), and perturbed barrier integrity (Figure 2D,E), that all contribute to the pathology involved in the high mortality [20,46]. As the associations between sympathetic activation, endothelial glycocalyx shedding, and 30-day mortality were both independent of injury severity, inherent variations in both of these biological systems appear to be involved in the heterogeneity in outcome between shocked trauma patients with similar clinical presentations [46,48,49,50]. The main aim here is, therefore, to review the experimental evidence of an interplay between the SNS and the microvascular endothelium leading to SHINE that contributes to the high mortality at the molecular and cellular levels in patients experiencing trauma with shock. To provide an overview of the effects of traumatic shock-induced SNS activity on ECs, literature searches were conducted on PubMed and Google Scholar using the search string: (“traumatic shock” or “sympathetic nervous system” or “catecholamines”) and “endothelial cells”. Reviews published within the last 5 years were selected and combined with pivotal older reviews. Based on this literature, molecular pathways connecting traumatic shock to endothelial glycocalyx damage and increased barrier permeability were synthesized into a molecular regulatory network. This was followed by a targeted search for experimental reports supporting each interaction in the network, and any interaction based on conflicting evidence was excluded.

## 2. Trauma, Excess Catecholamine Signaling and SHINE

The release of excess levels of catecholamines, secondary to trauma with shock, has profound effects on ECs mediated by their signalling through the adrenergic and dopaminergic receptors [50,51]. A dose-dependent correlation has been observed clinically and experimentally between catecholamine levels and loss of endothelial glycocalyx, cleavage of TM with impairment of the protein C system, and impaired endothelial barrier permeability [17,46,48,52].

### 2.1. Excess Catecholamine Levels Increase Oxidative Stress, Resulting in Glycocalyx Damage and Cleavage of TM from the Endothelial Cell Membrane

**Figure 3:**
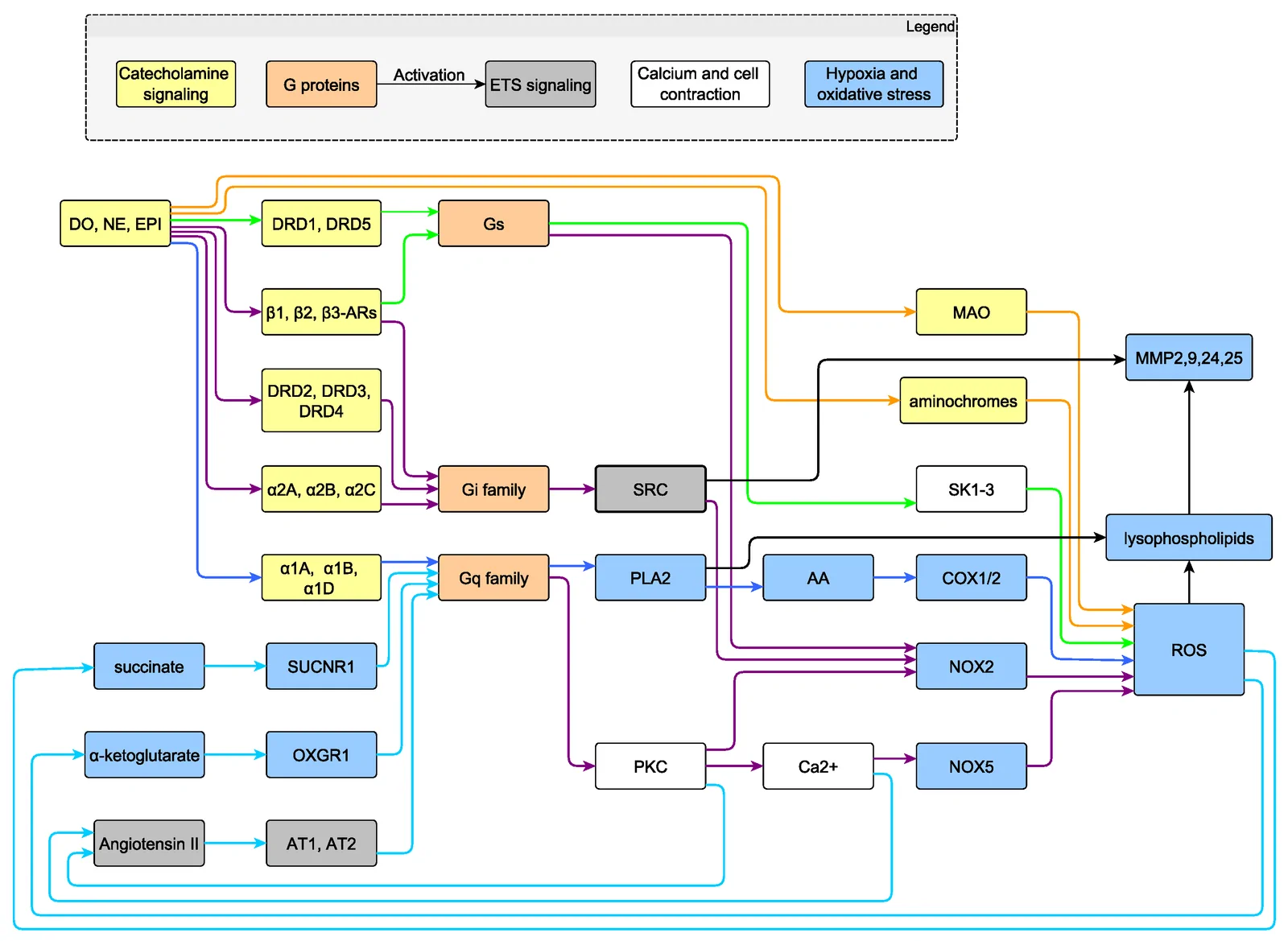
Catecholamines increase oxidative stress: The pathways are classified by the color of the arrows, (blue) Catecholamines bind guanine nucleotide-binding protein subunit αq/11 (Gq)-coupled α1 adrenergic receptors (ARs), which increase phospholipase A2 (PLA2) activity with arachidonic acid (AA) production. Many of the enzymes involved in AA metabolism, including cyclooxygenase-1 (COX1) and cyclooxygenase-2 (COX2), produce reactive oxygen species (ROS). (turquoise) Gq signaling is amplified through Angiotensin II, succinate, and α-ketoglutarate. (purple) Gi and Gq-coupled adrenergic and dopaminergic receptor signaling leads to the activation of NADPH oxidase 2 (NOX2) and NADPH oxidase 5 (NOX5), increasing ROS production. (green) Gs-coupled adrenergic and dopaminergic receptor signaling increases ROS production through small conductance calcium-activated potassium channel protein 1-3 (SK1-3). (orange) the catabolism of catecholamines in the mitochondria produces ROS, and (black) the non-receptor tyrosine kinase c-Src (SRC) and oxidized phospholipids activate matrix metalloproteinases 2, 9, 24, and 25 (MMP2, 9, 24, 25), resulting in glycocalyx damage.

Catecholamines increase the production of reactive oxygen species (ROS), including the superoxide ion, H_2_O_2_, and peroxynitrite, leading to oxidative stress. Some of the mechanisms through which catecholamines increase ROS production involve adrenergic and dopaminergic receptor signaling (Figure 3). α1 ARs are coupled to $G_{\alpha q/11}$, which activates the phospholipase A2 (${\mathrm{PLA}}_{2}$) [53]. The phospholipid hydrolysis catalyzed by ${\mathrm{PLA}}_{2}$ generates arachidonic acid (AA), and many of the enzymes involved in AA metabolism, such as the cyclooxygenases COX-1 and COX-2, generate ROS [32,54,55]. Additionally, α1 AR signaling produces angiotensin (Angiotensin II), which binds the $G_{\alpha q/11}$-coupled receptor AT1 and further increases AA metabolism and ROS synthesis [51]. Additionally, excess catecholamines, through adrenergic signaling, increase the activity of NADPH oxidases (NOX1, NOX2, NOX4, and NOX5), which produce the ROS superoxide ion and $H_{2}$$O_{2}$ [56]. $G_{\alpha q/11}$-coupled α1 ARs activate NOX5 through Angiotensin II and ${\mathrm{Ca}}^{2+}$ signaling [51,57]. Moreover, α1 ARs activate NOX2 through protein kinase C (PKC) [45], which phosphorylates the p47-phox subunit of the NOX2 complex, causing its translocation to the cell membrane where it is activated, resulting in increased superoxide production [58,59,60]. NOX2 is an important source of ROS in the arterial wall [61] that is present as a functional complex associated with the cytoskeleton in ECs [62]. Further, $G_{\alpha i/o}$-coupled α2 ARs [63] and β ARs [45] can also activate SRC, which phosphorylates the p47-phox subunit of the NOX2 complex and increases superoxide production [58,59,60]. Furthermore, excess catecholamines, such as those seen in shocked trauma patients with SHINE [64,65], can signal through the Gαs-coupled DRD1 and DRD5 receptors, which activate small conductance calcium-activated potassium (SK1-3) channels via PKA and NOX-derived ROS [66].

Excess catecholamines can also increase oxidative stress through mechanisms that are independent of adrenergic and dopaminergic receptors (Figure 3). ECs can absorb catecholamines [67], and once inside the EC, the catecholamines are transported to the mitochondria, where they are degraded by L-monoamine oxidase (MAO) and Catechol-O-methyltransferase (COMT). MAO catalyzes the oxidative deamination of catecholamines, producing hydrogen peroxide as a byproduct. Additionally, catecholamine degradation produces aminochromes, which are reduced to aminochrome semiquinones by complex I, resulting in the production of superoxide. Moreover, the increase in oxidative stress causes mitochondrial permeability transition pores to open, resulting in mitochondrial depolarization, which amplifies ROS [68]. In trauma patients with SHINE, the oxidative stress generated by the excess catecholamine levels causes succinate [69,70] and α-ketoglutarate [71] accumulation. Succinate binds SUCNR1 and α-ketoglutarate binds OXGR1, both activate $G_{\alpha q/11}$ [70], further increasing oxidative stress [58]. Additionally, after reperfusion, the accumulated succinate is reoxidized by succinate dehydrogenase, resulting in the production of more ROS [69].

Pivotally, excess catecholamine levels cause membrane lipid oxidation and activate ${\mathrm{PLA}}_{2}$, which preferentially cleaves oxidized lipids to produce lysophospholipids. This increases the interaction between the endothelial glycocalyx component syndecan-1 and the matrix metalloproteases MMP24 and MMP25, resulting in glycocalyx damage [72] (Figure 3). In alignment with this, experimentally high NE levels activate ${\mathrm{PLA}}_{2}$ through $G_{\alpha q/11}$, leading to the same HPAEC membrane reorganization that promotes the interactions of MMP24 and MMP25 with glycocalyx constituents as described for high succinate levels, suggesting a direct effect of high circulating catecholamine levels [72]. This is in alignment with experimental findings reporting a dose-dependent loss of glycocalyx, as measured by transmission electron microscopy upon the addition of catecholamines [50]. The oxidative stress, secondary to excess catecholamines, also increases the expression and activity of MMP2 and MMP9, contributing to glycocalyx shedding [73], as well as to the cleavage of TM from the EC membrane, impairing the protein C system anticoagulation [74].

### 2.2. Effects of Excess Catecholamine-Induced Glycocalyx Shedding and TM Cleavage on the Endothelial Cells

**Figure 4:**
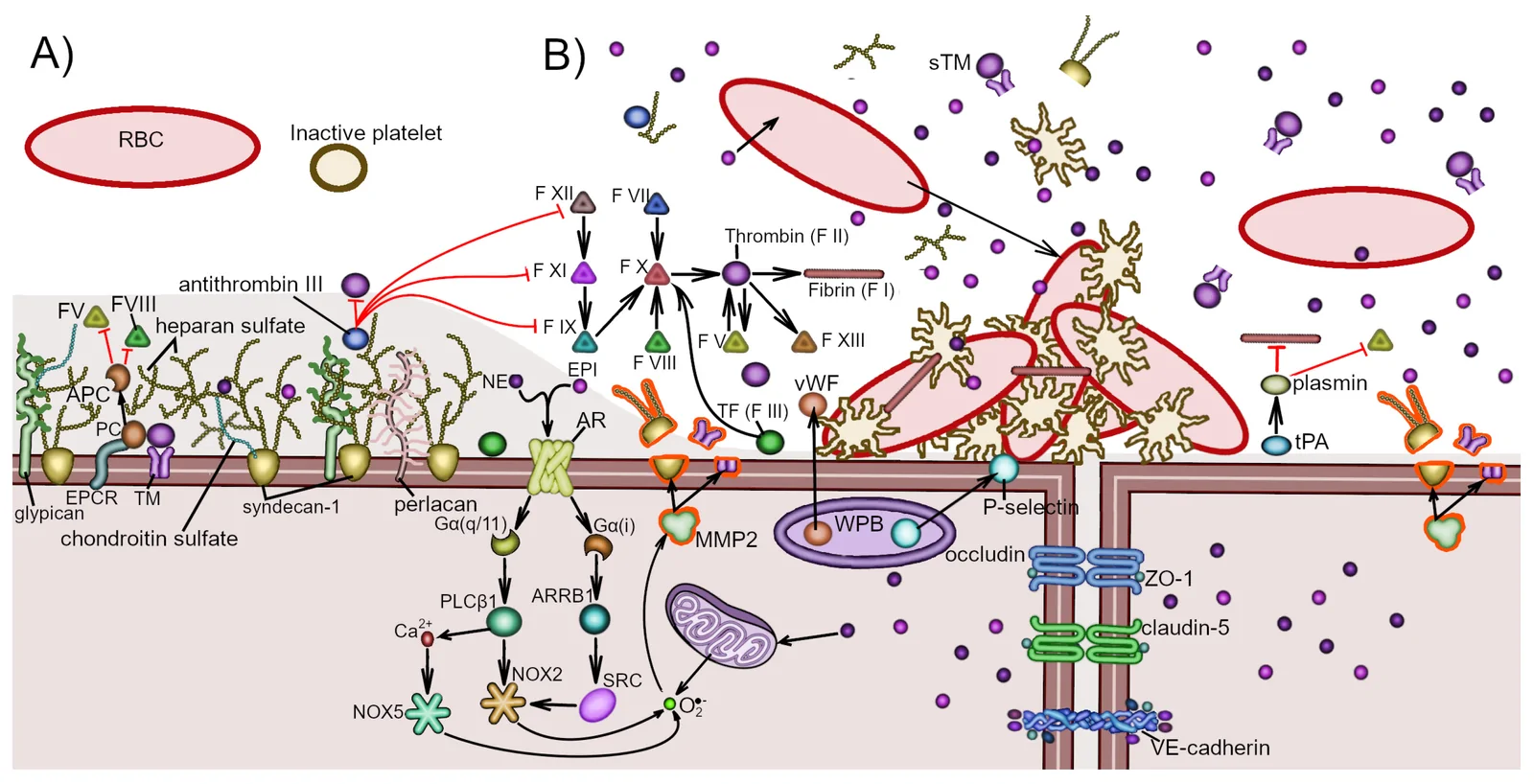
Catecholamine-induced oxidative stress causes glycocalyx damage: Black arrows represent activation, red blunt arrows represent inhibition, (**A**) Healthy endothelium and (**B**) traumatic shock-induced endotheliopathy. The consequences of glycocalyx, syndecan-1, -and thrombomodulin (TM) shedding (highlighted in orange) secondary to excess catecholamine levels are: 1. uncontrolled coagulation, platelet, leukocyte, and red blood cell (RBC) activation due to loss of antithrombin and activated protein C, which together with a prothrombotic EC surface leads to microvascular thrombosis and hypoxia, and 2. the development of trauma-induced coagulopathy due to an excess release of tissue plasminogen activator (tPA) from the chromaffin cells, where it is stored with epinephrine (EPI)/norepinephrine (NE), and from the endothelium. Excess tPA generates excess levels of plasmin, which both breaks down the clots (hyperfibrinolysis) and inactivates activated coagulation factor V (FVa) (hypocoagulation), resulting in treatment-refractory hemorrhagic shock.

Excess catecholamine levels result in the loss of glycocalyx constituents, including syndecans, glypicans, and perlecans, together with other glycosylated proteins, such as the hyaluronic acid receptor CD44 [75,76,77]. Hereby, PECAM1, selectins like P-Selectin, integrins, ICAMs, VCAMs, and other membrane-bound proteins are uncovered to the circulating blood, leading to the activation and adhesion of activated platelets and erythrocytes [75,77] (Figure 4B). Also, glycosaminoglycan (GAG) chains—hyaluronic acid, chondroitin sulfate, and heparan sulfate—are lost, further contributing to the interaction of negatively charged platelets and red blood cells (RBC) with the increasingly procoagulant endothelial surface [76,78] (Figure 4B). Loss of heparane sulphate is of particular importance for the trauma-induced pro-thrombotic endothelial surface since this impairs the anticoagulant effect of antithrombin III [79], which is a strong inhibitor of procoagulant enzymes like thrombin and activated factors IX, X, XI, and XII [80,81]. Furthermore, trauma-induced excess catecholamine signaling contributes to the exocytosis of Weibel-Palade Bodies from the endothelium, leading to the release of mainly vWF, P-selectin, and ANG2 and further contributing to a procoagulant state of the endothelium [82,83,84,85] (Figure 4B). Importantly, excess catecholamines also activate RBCs through β2-adrenergic receptor-induced adenyl cyclase (AC) activation [86,87]. The surface interactions of activated RBCs govern signaling between platelets and RBCs and also aid in the conversion of prothrombin to thrombin, a pivotal step in the generation of clots. Additionally, RBCs and platelets generate microparticles, which have been shown to reduce clotting time, ensuring faster clot generation. Finally, blood clot structure and maturation are dependent on the inclusion of RBCs in forming thrombi [88,89] (Figure 4B).

Furthermore, the excess catecholamine-induced oxidative stress upregulates MMPs to also cleave TM from the EC surface into its soluble form with profound effects on the EC phenotype [73,74]. TM is a pivotal part of the protein C anticoagulant system, and it has been reported that severely injured trauma patients with high catecholamine levels consistently also have increased levels of circulating soluble thrombomodulin (sTM), indicating its cleavage from the luminal endothelial surface [17,90,91]. In alignment with this, catecholamine levels have been reported to correlate with sTM levels in patients suffering from trauma [17] and in healthy volunteers exposed to stress by immersion in hot and cold water, potentially involving the PKA pathway [92] Similarly, a strong correlation between sTM and plasma catecholamines was found in healthy rats exposed to hypothermic stress and rewarming, possibly involving heat shock transcription factor 1 (HSF1) [93] Loss of TM contributes significantly to a pro-thrombotic endothelial surface in trauma patients by impairing the generation of activated protein C (APC), which impairs the inactivation of FV and FVIII and, ultimately, thrombin, leading to an uncontrolled coagulation activation on the surface of the microvascular ECs [94] (Figure 4B). Also, loss of EC TM increases thrombin-PAR1 signaling and vWF secretion, leading to ectopic platelet activation, further contributing to a prothrombotic state [95]. In trauma patients with SHINE, the above-described effects of excess catecholamine release occur systemically throughout the microvasculature, and this contributes to the high mortality observed here [18,48,96]. The uncontrolled coagulation activation, together with the activation of the platelets and RBCs, leads to the development of systemic capillary thromboses with hypoxia of the underlying tissues as well as exacerbation of the SNS activation and catecholamine release, creating a vicious circle and, ultimately, organ failure and death [19,48]. A consistent observation in trauma patients with SHINE is that they also present with trauma-induced coagulopathy (TIC) [46]. TIC is characterized by severe hypocoagulability and severe hyperfibrinolysis, which are associated with increased transfusion requirements, exsanguinations, and severalfold increased 28-day mortality when compared to non-TIC patients [97,98]. Trauma patients with SHINE, characterized by excess catecholamine levels, have significantly lower levels of FII, FV, FIX, FX, and FXI than those without SHINE [65,99]. The most discriminative feature concerning hypocoagulation between trauma patients with and without SHINE was low FV, and in alignment with this, a recent study in 1285 trauma patients with hemorrhagic shock reported that low FV was associated with increased transfusion requirements and increased 28-day mortality, confirming its clinical relevance [99] (Figure 4C). Concerning hyperfibrinolysis, catecholamine release directly impacts the severity of this condition in trauma patients with SHINE, as tissue plasminogen activator (tPA) is packaged together with catecholamines in the storage vesicles of chromaffin cells, in addition to being released by ECs [100,101,102,103]. tPA converts plasminogen into active plasmin, which dissolves the fibrin mesh of the clot. Consequently, excess sympathetic activation, along with the release of excess catecholamines and tPA, is correlated with the degree of hyperfibrinolysis experienced by trauma patients with SHINE [104]. Excess tPA release may also be involved in the hypocoagulation of TIC observed in SHINE patients, as the high tPA activates excess plasmin, which is a promiscuous protein that also has been reported to inactivate FV, ultimately preventing thrombin generation [65,91] (Figure 4C).

### 2.3. Excess Catecholamines Increase Endothelial Barrier Permeability by Modulating Calcium and cAMP Signaling

**Figure 5:**
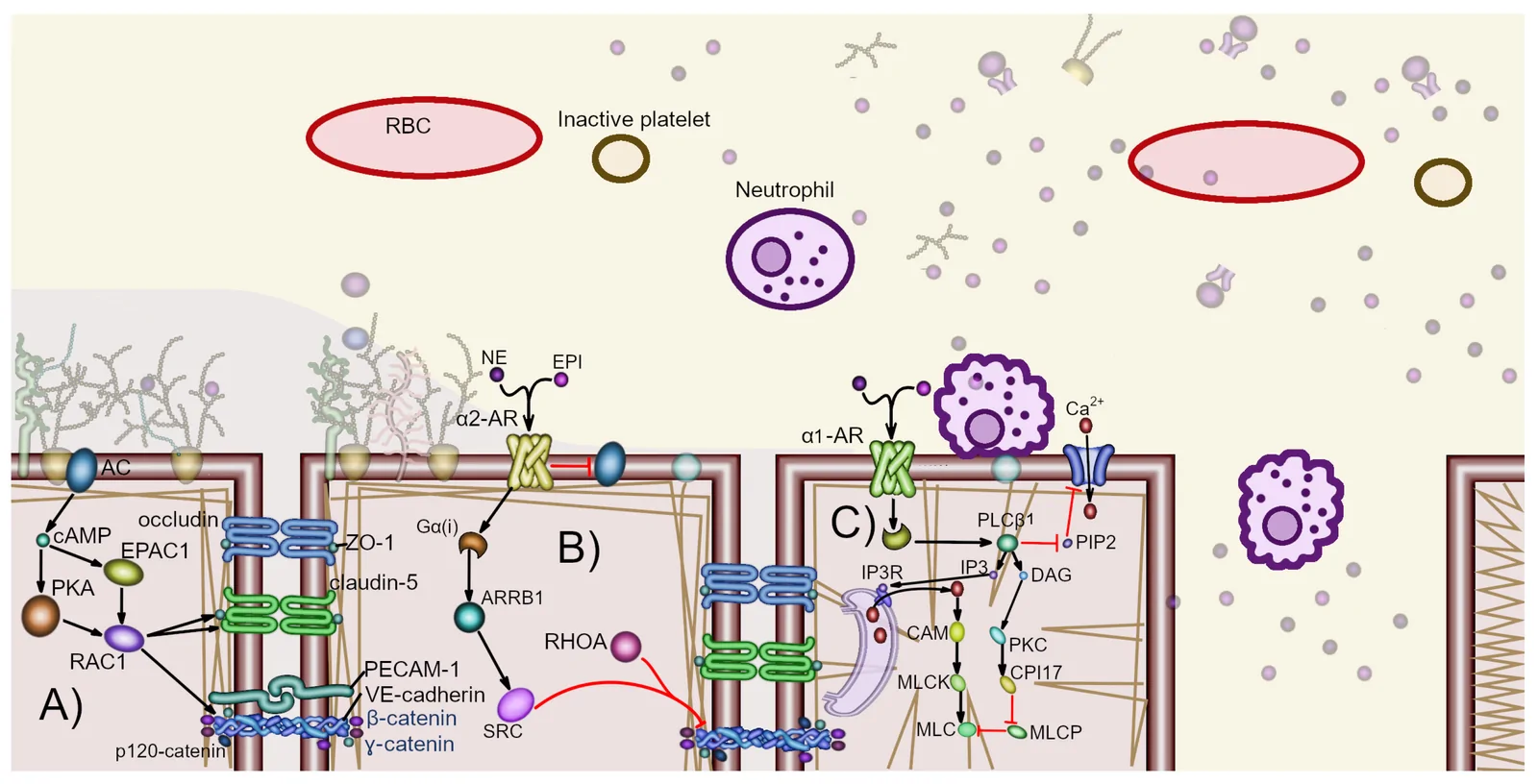
Sympathetic nervous system activation with catecholamine release increases endothelial barrier permeability: Black arrows represent activation, red blunt arrows represent inhibition, (**A**) A healthy quiescent EC where cyclic adenosine monophosphate (cAMP) stabilizes tight junctions (TJs) and adherence junctions (AJs) through Ras-related C3 botulinum toxin substrate 1 (RAC1), (**B**) α2-adrenergic receptor (AR) signaling destabilizes AJs and TJs, (**C**) α1-AR signaling causes endothelial cell contraction, which, combined with weakened interendothelial junctions, increases endothelial barrier permeability and diapedesis.

**Figure 6:**
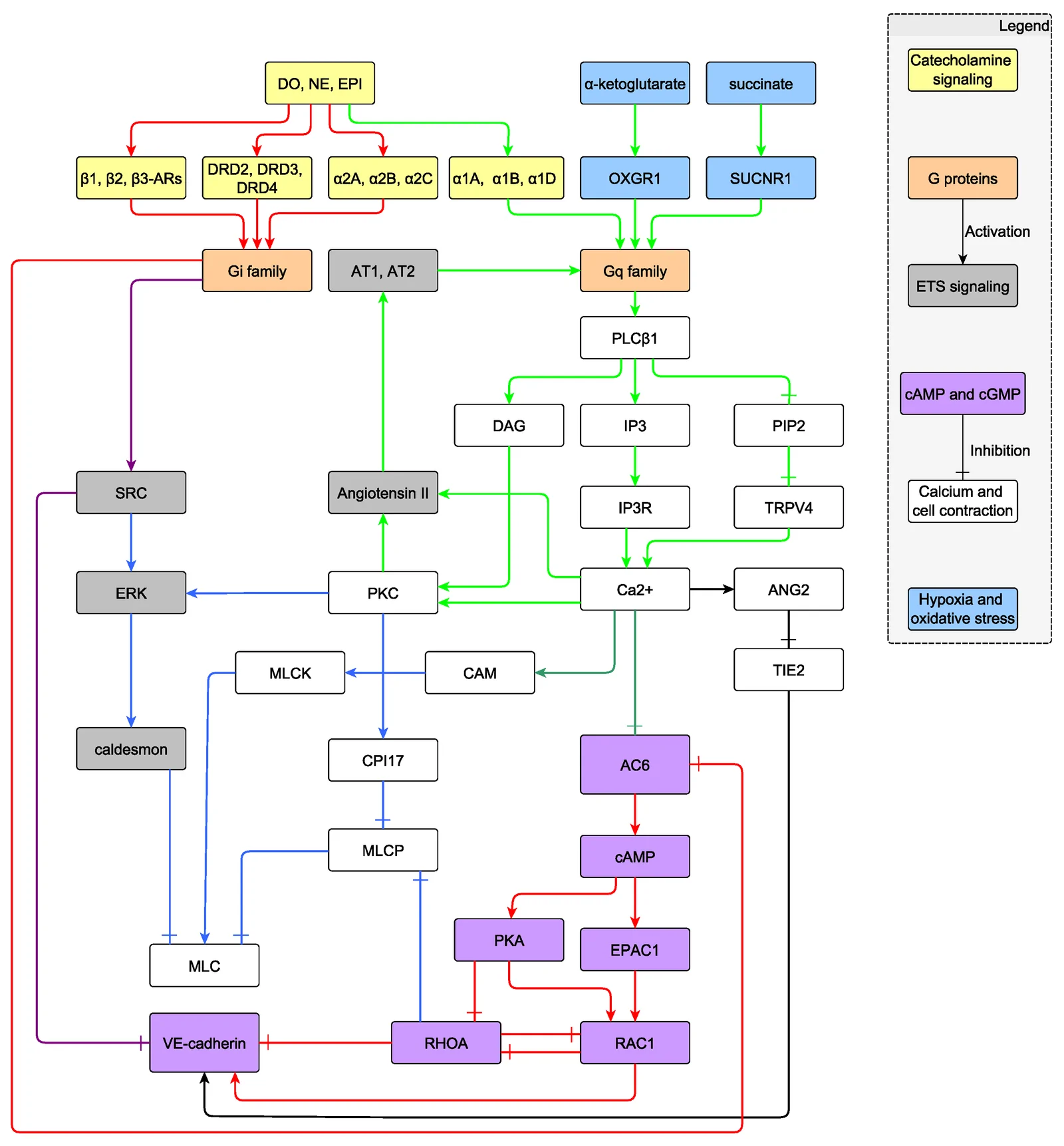
High catecholamin levels increase endothelial barrier permeability: The pathways are classified by the color of the arrows, (red) Guanine nucleotide-binding protein subunit αi/o (Gi)-coupled adrenergic and dopaminergic receptor signaling inhibits adenylyl cyclase 6 (AC6), reducing cyclic adenosine monophosphate (cAMP) production and destabilizing endothelial tight junctions (TJs) and adherence junctions (AJs). (purple) Additionally, Gi signaling activates the non-receptor tyrosine kinase c-Src (SRC), which phosphorylates junctional proteins including vascular endothelial-cadherin (VE-cadherin), further destabilizing endothelial TJs and AJs. (green) Gq-coupled α1-adrenergic receptors and angiotensin II receptor type 1 (AT1) signaling increases the cytoplasmic ${\mathrm{Ca}}^{2+}$ concentration through phospholipase C β 1 (PLCβ1). (dark green) ${\mathrm{Ca}}^{2+}$ further decreases cAMP production, destabilizing AJs and TJs, and increasing the activity of Ras homolog family member A (RHOA). (blue) ${\mathrm{Ca}}^{2+}$ and RHOA cause the formation of actin stress fibers and increase myosin light chain (MLC) activity, resulting in endothelial cell (EC) contraction. (black) ${\mathrm{Ca}}^{2+}$ also increases the secretion of (blue) angiopoietin-2 (ANG2), which inhibits tyrosine kinase with immunoglobulin and epidermal growth factor homology domains (TIE2) signaling, resulting in less stable AJs. The combination of junction destabilization and EC contraction increases endothelial barrier permeability.

ECs form a selectively permeable barrier by attaching through adherens junctions (AJs) and tight junctions (TJs), which are connected to cytoskeletal components like actin through catenins and zona occludens (ZO-1) proteins, providing intracellular anchorage. AJs are composed of VE-cadherin, which mediates EC adhesion through inter-endothelial trans-dimerization [105]. Additionally, PECAM-1 attaches to AJs, where PECAM-1-PECAM-1 homophilic interactions enhance barrier function and contribute to mechanotransduction [106,107]. TJs are formed by adhesion proteins like claudins and occludin, which dimerize, bridging the intercellular space [108] (Figure 5A). Excess catecholamines increase endothelial barrier permeability by simultaneously reducing the density of AJs and TJs and stimulating EC contraction (Figure 6).

Trauma-induced catecholamine signaling increases vascular permeability by regulating adenylyl cyclases (ACs) that are the main enzymes that produce cyclic adenosine $3^{\prime }$,$5^{\prime }$-monophosphate (cAMP), which preserves AJs and TJs, and prevents the formation of the actin stress fibers that are needed for EC contraction [109] (Figure 5A). The dopaminergic receptors DRD2, DRD3, and DRD4 [110], and α2 and β ARs [111] inhibit transmembrane AC activity through $G_{\alpha i/0}$ signaling, which also activates SRC [63]. Then, SRC destabilizes VE-cadherin complexes, weakening AJs [112] (Figure 6 and Figure 5B).

One of the main mechanisms that allows ECs to integrate intracellular and extracellular cues is a transient increase in cytoplasmic ${\mathrm{Ca}}^{2+}$ concentration [113]. NE and EPI cause EC contraction and increase endothelial barrier permeability by activating $G_{\alpha q/11}$ signaling through the α1 ARs ADRA1A, ADRA1B, and ADRA1D, which activate the phospholipase PLCβ1 [53,68] (Figure 6). At least two molecular mechanisms enable PLC to increase the cytoplasmic ${\mathrm{Ca}}^{2+}$ concentration. PLC catalyzes the reaction that cleaves phosphatidylinositol 4,5-bisphosphate (PIP2) to produce inositol 1,4,5-trisphosphate (IP3) and diacyl glycerol (DAG). Subsequently, IP3 binds IP3R and causes the release of ${\mathrm{Ca}}^{2+}$ from internal reserves in the endoplasmic reticulum [114]. Additionally, by cleaving PIP2, PLC promotes transient receptor potential vanilloid 4 (TRPV4) channel activity, increasing ${\mathrm{Ca}}^{2+}$ entry from the blood plasma into the cytosol [115].

The ${\mathrm{Ca}}^{2+}$ pulses caused by excess catecholamines can reduce the activity of ACs either directly or through CAM, calcineurin, and PKC, where ${\mathrm{Ca}}^{2+}$ directly inhibits AC5 and AC6 [53,116]. Further, even a submicromolar increase in ${\mathrm{Ca}}^{2+}$ concentration near the cell membrane can inhibit AC6, decreasing the concentration of cAMP near the cell membrane and increasing the permeability of the endothelial barrier [117] (Figure 6). The effect of low sub-membrane cAMP on junctional integrity includes preventing PKA and EPAC1-mediated RAC1 activation, which allows increased phosphorylation of VE-cadherin, β-catenin, and p120-catenin. This weakens the cadherin-actin complex anchorage, causing junctions to become “leaky”. Low PKA activity also promotes actin stress fiber formation and disinhibits RHOA and reduces MLCP activity [109]. Additionally, the elevated cytoplasmic ${\mathrm{Ca}}^{2+}$ concentration activates calmodulin (CAM) and MLCK [118], and DAG activates PKC and CPI17, which further inhibits MLCP [119]. The activation of MLCK and inhibition of MLCP increase MLC phosphorylation and EC contraction. PKC also increases ERK activity [120], which inhibits caldesmon and promotes cytoskeletal remodeling and actin and MLC-mediated EC contraction [121,122]. Weakened AJs and TJs, combined with stress fiber formation and actomyosin contraction, open interendothelial gaps that disrupt endothelial barrier function (Figure 5C).

The high ${\mathrm{Ca}}^{2+}$ levels associated with excess catecholamines can cause Weibel–Palade bodies (WPBs) to fuse with the EC membrane and release ANG2 [123], resulting in increased endothelial barrier permeability.

ANG2 blocks ANG1 from binding to TIE2 at the endothelial cell-cell contacts [124]. The inhibition of TIE2 signaling by ANG2 results in the disruption of the endothelial barrier by activating β1-integrin [125] and by breaking down VE-cadherin [126], leading to the formation of paracellular gaps and increased endothelial barrier permeability. In alignment with this, in a porcine model of hemorrhagic shock, high catecholamine levels were observed together with an increase in ANG2 to ANG1 ratio and high syndecan-1 when the animals were in shock [127], likely because of the PLC-γ1 and PKA-induced WPB exocytosis [128], followed by ANG2 inhibition of ANG1 and TIE2 signaling, increasing endothelial barrier permeability [129].

### 2.4. Effects of Excess Catecholamine-Induced Endothelial Barrier Permeability

A unique and pivotal property of the endothelial cell–cell interaction is its gatekeeper function, which determines the fraction of mounted catecholamines, pro-inflammatory mediators, activated immune cells, and damage-associated molecular patterns (DAMPs) that reach the underlying tissues, including the vital organs, secondary to the injurious insult [41]. Furthermore, increased barrier permeability, as observed in shocked trauma patients with SHINE, also leads to profound extravasation with two detrimental consequences. Firstly, the extravasation of fluid leads to increased pressure in the underlying tissues, which impairs oxygen delivery to the cells of vital organs prone to hypoxia [130] (Figure 5C). Secondly, the loss of intravascular volume leads to sustained hypotensive shock and, collectively, both hypoxia and hypotension sustain and exacerbate SNS activation with the release of high levels of catecholamines, creating a vicious circle contributing to the increased mortality observed [29,30,31,33,34]. Concerning the mounted proinflammatory response to trauma with shock that traverses the disintegrated endothelial barrier in patients with SHINE, this is counteracted by the excess release of catecholamines into the systemic circulation through sympathoadrenal activation and adrenergic receptor signaling, as well as into specific tissue beds such as thymus, spleen, lymph nodes, bone marrow, heart, blood vessels, bronchi, and gastrointestinal tract (GI) through noradrenergic discharge from sympathetic nerve terminals [25,131,132,133,134,135,136]. This constitutes a crucial component of the neuroimmune communication system as all immune cells except T helper 2 (Th2) cells express α1, α2, and β ARs [137]. The profound effects on the immune system of excess catecholamines in SHINE levels are illustrated by that they reduce the cytotoxicity of CD8 T lymphocytes, decrease IL12 production of dendritic cells, inhibit the formation of neutrophil extracellular traps (NETS), suppress the cytotoxicity and migration of natural killer (NK) cells, reduce the production of antibodies by B lymphocytes impairing adaptive immunity, decrease the phagocytic activity of monocytes, and suppress proinflammatory M1 polarization and promote anti-inflammatory M2 behavior of macrophages [137,138,139,140,141]. Additionally, macrophages, neutrophils, and T and B lymphocytes can synthesize, store, and release catecholamines de novo, acting in an autocrine and paracrine manner to modulate the proinflammatory response locally [135,137,140]. Collectively, in trauma patients with SHINE and excess catecholamine release, this results in an overwhelming impairment of immune competence, both of the circulating immune cells as well as those that have crossed the damaged endothelial barrier into the underlying tissues, contributing to the high mortality observed in these patients [135].

## 3. Discussion

### 3.1. The Experimental Evidence Supporting the Connection Between SNS Activation, Endotheliopathy, MOF, Coagulopathy, and Mortality

The main finding of this review is that extensive experimental evidence supports that SNS activation and the accompanying excess catecholamine release and downstream signaling secondary to trauma and hemorrhagic shock have profound effects on the microvascular ECs, contributing to the dreaded complications of SHINE in a subpopulation of patients. This finding builds on the unprecedented success of research in molecular medicine, identifying vital components and pathways that contribute to the microvascular endothelial cellular phenotype in health and under stress, corroborated by more than 23,000 PubMed hits using these terms as search criteria (July 2026) [142,143,144]. Recent breakthroughs in the field that strengthen the connection between SNS activity, SHINE, MOF, and coagulopathy include the contribution of dopaminergic receptor signaling to the development of SHINE [66,145], the importance of calcium import for trauma-induced endothelial permeability [146], the role of Angiotensin II [51,147] and succinate [72] in amplifying $G_{\alpha q}$ signaling, contributing to glycocalyx damage and increased permeability, the protective [148] or nocive [149] potential of β-AR signaling, the importance of laminar flow for endothelial barrier function [150], and the importance of TM [151] and FV [91,99] in preventing coagulopathy and restoring hemostasis. Unfortunately, molecular medicine research also constitutes the main limitation of the present manuscript, as these experiments are conducted in static cell cultures or animal experiments due to the difficulty in obtaining adequate viable capillary samples of human origin [152] (Table 1). Collectively, the limitations of these in vitro approaches are that the EC samples undergo extensive manipulation and processing steps that perturb their native EC phenotype [152]. Concerning EC culture studies, ECs are notably sensitive to mechanical forces—static growth conditions, a growth medium that has viscoelastic properties that are very different from those of blood, and the use of materials that are very hard compared to the tissue that normally surrounds ECs—substantially change the physical forces modulating EC behavior [152,153]. Similarly, animal models have allowed us to gain crucial insights about traumatic shock [154,155]. However, evolution has also affected the response to traumatic shock, resulting in important differences that prevent us from answering important questions about the human response to trauma with shock based only on animal model research. A further pivotal limitation of the published reports, to date, is that they investigate isolated molecular constituents, targets, and pathways, and therefore, the contribution of other pivotal pathways and biological systems, including their redundance, on the EC phenotype under in vivo conditions is lacking, complicating the interpretation of the results in a human context [154,156,157].

The effect of catecholamine signaling on ROS generation, where the ability of EPI and NE to bind AR-α, AR-β, and dopaminergic receptors to activate GPCRs is supported by molecular evidence and EC culture experimental results (Table 1). Results from human EC cultures and experiments using mice (Table 1) trace molecular pathways that link GPCR activity to ROS generation [45,51,53,57,60,66]. Based on human EC culture experimental results, ROS generation causes structural damage to the glycocalyx directly by acting on heparan sulphate with shedding of the glycosaminoglycan chains from the cell surface [213]. ROS also acts as signaling molecules that upregulate and activate enzymes like heparinase, hyaluronidase, and MMPs that damage the glycocalyx [214,215]. Concerning MMP2 and MMP9, specifically, experiments on rats support that ROS increase their expression and activity [216]. Additionally, based on human microvascular EC cultures and experiments on mice, ROS-activated MMP2 and MMP9 contribute to syndecan-1 shedding, and to the cleavage of TM from the EC membrane [73,217], the hallmarks of SHINE as observed clinically in shocked trauma patients [74]. Glycocalyx loss and the impairment of the protein C anticoagulant system are detrimental for the microvascular EC phenotype systemically, as it leaves its luminal surface largely unprotected. The severely prothrombotic surface leads to the activation and adhesion of platelets, leukocytes, and RBCs [76,78], which, together with uncontrolled coagulation activation due to lack of antithrombin and activated protein C, result in microvascular thromboses throughout the capillary networks and hypoxia of the underlying tissues; this has been corroborated on observational clinical studies [80,94,96].

Similarly, results from experiments on rats and bovines (Table 1) support that excess catecholamine signaling, through $G_{\alpha q/11}$-coupled endothelial ARs, activates phospholipase PLCβ1 [53]. Human molecular evidence suggests (Table 1) that PLCβ1 stimulates the release of ${\mathrm{Ca}}^{2+}$ from the endoplasmic reticulum [114], and results from experiments on mice support the notion that PLCβ1 increases ${\mathrm{Ca}}^{2+}$ entry from the blood plasma to the cytosol [115]. Rat and mice experiments (Table 1) suggest that this transient increase in cytosolic ${\mathrm{Ca}}^{2+}$ inhibits AC6, decreasing the level of cAMP near the cell membrane [117]. Low cAMP levels reduce the activity of RAC1 and increase the activity of RHOA, destabilizing endothelial AJs and TJs, reducing VE-cadherin accumulation at the cell junctions, and weakening the cadherin-actin complex [109]. This is supported by experiments on mice (Table 1). Additionally, based on EC cultures without flow (Table 1), ${\mathrm{Ca}}^{2+}$ increases MLC activity and RHOA-induced stress fiber formation [121,122], leading to actomyosin contraction pulling the cells apart and increased barrier permeability [146]. Collectively, these structural changes of the microvascular ECs lead to progressive hypoxia of the underlying tissues due to systemic microvascular thrombosis and the increased tissue pressure secondary to extravasation of fluid from the vascular compartment, resulting in cellular hypoxia as well as sustained sympathetic activation with catecholamine release and high mortality, as observed in clinical studies [17,46,48]. The increased barrier permeability further provides unhindered access of excess levels of proinflammatory mediators, DAMPs, catecholamines, and more to the underlying damaged and vulnerable tissues. Additionally, excessively activated leukocytes with impaired immune competence have free access through the leaky endothelial barrier to the vital organs of trauma patients with SHINE, prone to the high mortality observed [44,135].

### 3.2. The Interplay Between SNS Activity, Hyperfibrinolysis, and Hypocoagulation

TIC is a dreaded complication in trauma patients with hemorrhagic shock and is associated with severalfold increased mortality [97,98]. Despite the introduction of hemostatic resuscitation, the high mortality of the most critically ill with TIC remains [65]. The hallmarks of TIC (hypocoagulation and hyperfibrinolysis) are observed in the 20% of shocked trauma patients presenting with SHINE. The interplay between the SNS and the ECs is pivotal for the hyperfibrinolysis of TIC as the catecholamines cause ECs to release tPA [101,102]. Furthermore, tPA is also stored in vesicles with catecholamines in the chromaffin cells and is co-released in response to sympathetic activation, which in trauma patients with SHINE results in excess levels of tPA [100]. Excess tPA levels lead to excess plasmin generation, which dissolves the clot, leading to excess bleeding [103]. Concerning the hypocoagulation of TIC, several potential mechanisms have been proposed, such as increased levels of activated protein C [218,219], consumptive coagulopathy [10], and endogenous heparinization [220]. Recently, we reported that low FV was the second most discriminating feature concerning hypocoagulation between trauma patients with SHINE, as evaluated by the EPI, syndecan-1, and sTM, and those without [65]. FVa is pivotal for intact thrombin generation necessary for the development of a strong clot and the achievement of hemostasis. In alignment with this, a study of 1285 hemorrhaging trauma patients reported that those in the lowest FV quartile had higher transfusion requirements and higher 28-day mortality [99]. This indicates that either the FV levels in the currently used plasma products are too low to reverse this condition or that other mechanisms are involved. We recently suggested that the catecholamine-induced excess tPA release leads to excess generation of the pleiotropic enzyme plasmin, which also has been shown to inactivate FVa [91]. This could explain why the administered plasma does not reverse TIC, warranting further investigation. A consistent observation in shocked trauma patients suffering the same injury severity is that they present significantly different levels of SNS activation, as measured by EPI [17,19,46], and this correlates both with the severity of endotheliopathy and the 30-day mortality [18,50,221]. This suggests an inherent patient-specific effect related to the SNS contributing to the high mortality [17,18,19]. This is in alignment with experimental studies demonstrating significant interpersonal variation in how the sympathetic nervous system (SNS) is structured, regulated, and reacts to stress or stimuli. This influences individual differences in physiological responses, health outcomes, and risk for disease, including the development of SHINE [222,223,224,225,226,227,228]. An extreme example of the heterogeneity in sympathetic activation and catecholamine release is Takatsubo´s cardiomyopathy, where certain individuals react with an overwhelming sympathetic activation with the release of excess levels of catecholamines as a response to emotional stress, resulting in acute cardiac failure, “broken heart”, and high mortality [17,46]. Whether this complication also develops in certain trauma patients with SHINE is currently unknown. Similarly, experimental studies of heterogeneity in catecholamine release between individuals show that it is significant and driven by a combination of genetic, environmental, and physiological factors [229,230,231,232]. It has further been reported that similar levels of circulating catecholamines are observed in shocked trauma patients with significantly different severities of glycocalyx shedding and sTM cleavage from the EC membrane, both correlated with patient outcome, suggesting that significant interpersonal heterogeneity in the microvascular ECs response to a given level of catecholamines also exists. This is corroborated by experimental data reporting that the microvascular ECs gene expression, molecular profiles, and functional behaviors vary between individuals, influenced by both genetic factors and factors like age, sex, and health status [233,234,235,236,237,238,239,240].

### 3.3. Priorities for Future Translational and Clinical Research

**Figure 7:**
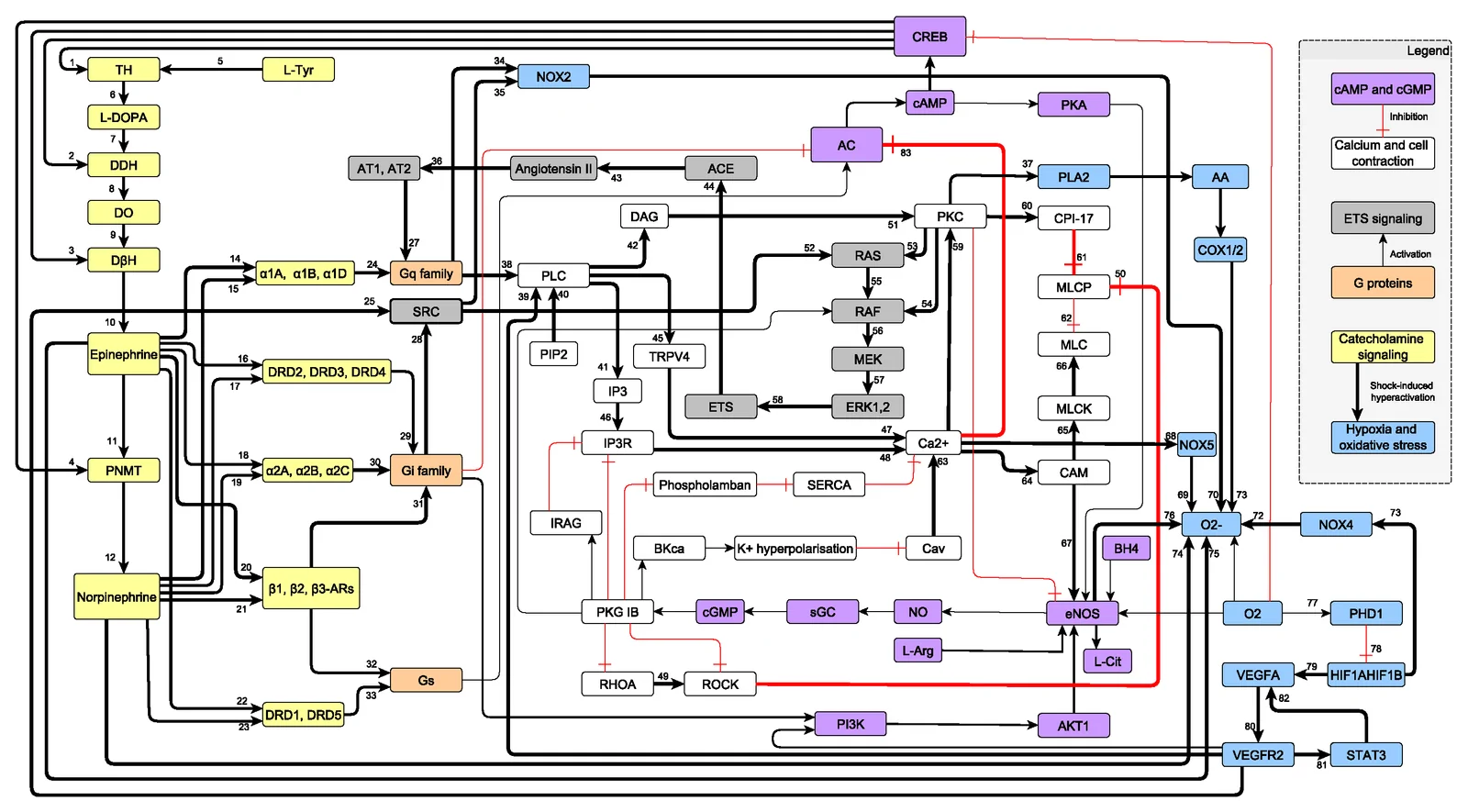
Endothelial catecholamine signaling: Trauma-induced elevated catecholamine levels increase oxidative stress, hypoxia, and calcium signaling, resulting in glycocalyx damage, EC contraction, and increased endothelial barrier permeability. In yellow, the metabolites and enzymes involved in catecholamine synthesis, the catecholamines, dopaminergic, and adrenergic receptors are shown. In orange, families of guanine nucleotide-binding (G) proteins. In gray, rat sarcoma virus (RAS), E26 transformation-specific (ETS), and angiotensin signaling. In white molecules involved in calcium signaling and endothelial cell (EC) contraction. In purple, molecules involved in cyclic adenosine monophosphate (cAMP) and nitric oxide (NO) production, and EC survival. In blue, molecules involved in hypoxia and oxidative stress. An interactive version of this figure is included as Supplementary Material.

Many of the molecular pathways that connect SNS activation with catecholamine release to glycocalyx damage and increased endothelial barrier permeability, likely contributing to MOF, TIC, and mortality, include molecular interactions that are supported only by experiments on animal models, experiments on EC cultures grown without flow, or cultures of non-endothelial human cells (Table 1). It would be important to corroborate these molecular interactions through observational clinical studies, or at least using human pulmonary, coronary, and cerebral microvascular ECs exposed to laminar flow and perturbed flow. Trauma-induced SNS activity with high levels of catecholamine release contributes to coagulopathy and edema through the previously described molecular pathways that converge on calcium import, ROS generation, junction destabilization, and MLC activation, forming a complex molecular regulatory network (Figure 7). The heterogeneity in the structure and multistability of this network, observed between patients, contributes to the variation in endothelial trauma response and warrants further research using computational modeling methods. Genetic or epigenetic variation in adrenergic receptors or endothelial signaling pathways may partially explain why patients with apparently similar injury severities develop markedly different degrees of endotheliopathy, and this phenomenon represents a future avenue for precision medicine approaches in trauma care. Advancing in this direction would require clinical observational research targeting the differences in function and expression of AT1, ARs, dopaminergic receptors, TRPV4, and other pivotal molecules between patients in the microvascular ECs of critical organs, including the lungs, heart, and brain. Understanding the differences in promoter accessibility and activity, combined with relevant micro and long noncoding RNA activity, would potentially predict the degree of traumatic injury-induced endotheliopathy, based on the clinical history of patients, and hopefully plan more effective treatment.

### 3.4. Important Unanswered Questions

Under which conditions do human microvascular ECs produce catecholamines during the traumatic shock response?

Which of the molecular mechanisms that have been observed only in the ECs of animal models, such as the activation of SRC by α2-AR-induced Gi signaling, are conserved in humans?

Given that β-ARs are highly expressed in ECs, which mechanism causes them to activate $G_{\alpha i}$ instead of $G_{\alpha s}$ signaling in microvascular ECs, PKA-mediated phosphorylation, ${\mathrm{Ca}}^{2+}$-mediated, or glycocalyx damage-induced positive membrane charge? How important is this during the EC response to traumatic shock?

The PKC/ERK/ETS/EGR1/ACE signaling pathway connects catecholamine signaling to Angiotensin II production, forming a feedback mechanism that amplifies barrier dysfunction. In several human, non-endothelial cell types, the ETS transcription factor ERK-activated ELK-1 activates EGR1 during the unfolded protein response. Is ELK-1 also involved in the endothelial response to traumatic shock?

## 4. Conclusions

Collectively, the reviewed data support, but do not yet prove, that the fight-or-flight response to traumatic shock also involves an interplay between the SNS and the microvascular endothelium, aiming to limit hemorrhage and resolve tissue damage. Clinical evidence suggests that due to the inherent heterogeneity of both the SNS and the microvascular ECs, a subpopulation of shocked trauma patients develops an adverse fight-or-flight response resulting in SHINE, independent of injury severity, with TIC, microvascular thrombosis, capillary leakage, and impaired immune competence [4]. The available evidence supports, but does not yet prove, that SHINE is a biologically plausible and potentially clinically relevant framework and causal mechanism that connects trauma-induced SNS activity with catecholamine release to MOF, TIC, and immune dysregulation, contributing to the high mortality observed.

## Figures and Tables

**Table 1:** Experimental evidence supporting the molecular pathways underlying the endotheliopathy. Abbreviations: AC 6, adenylyl cyclase 6; ACE, angiotensin-converting enzyme; AR, adrenergic receptor; AT-1, angiotensin II receptor type 1; CAM, calmodulin; cAMP, cyclic adenosine monophosphate; CKI-17, PKC-potentiated inhibitory protein for PP1; COX, cyclooxygenase; CREB, cAMP response element-binding protein; DAG, diacylglycerol; DβH, dopamine β-hydroxylase; DDH, dopa decarboxylase; DRD, dopamine receptor; EC, endothelial cell; EGR1, early growth response 1; ELK1, ETS transcription factor ELK1; eNOS, endothelial nitric oxide synthase; EPI, epinephrine; ERK, extracellular signal-regulated kinase; HIF1/HIF1A, hypoxia-inducible factor 1/1-alpha; HUVEC, human umbilical vein endothelial cell; IP3, inositol 1,4,5-trisphosphate; IP3R, inositol 1,4,5-trisphosphate receptor; MEK, mitogen-activated protein kinase kinase; MLC, myosin light chain; MLCK, myosin light chain kinase; MLCP, myosin light chain phosphatase; NE, norepinephrine; NOX, NADPH oxidase; PHD1, prolyl hydroxylase domain-containing protein 1; PIP2, phosphatidylinositol 4,5-bisphosphate; PKA, protein kinase A; PKC, protein kinase C; PLC, phospholipase C; PLA2, phospholipase A2; PNMT, phenylethanolamine N-methyltransferase; RAF, rapidly accelerated fibrosarcoma kinase; RAS, rat sarcoma GTPase; RHOA, ras homolog family member A; ROCK, Rho-associated protein kinase; ROS, reactive oxygen species; SNS, sympathetic nervous system; SRC, nonreceptor tyrosine kinase c-Src; STAT3, signal transducer and activator of transcription 3; TH, tyrosine hydroxylase; TRPV4, transient receptor potential vanilloid 4; TSAd, T-cell-specific adaptor protein; VEGFA, vascular endothelial growth factor A; VEGFR2, vascular endothelial growth factor receptor 2.

Evidence	Effect
Clinical	COX1 and COX2 increase oxidative stress [158], mitochondrial catecholamine metabolism increases oxidative stress [159]
EC cultures without flow	CREB increases TH, DDH, DβH, and PNMT transcription in bovine aortic ECs, and HUVECs [110,160], EPI increases ROS generation through DRD1 and DRD5 [66], Angiotensin II -activated AT-1 activates PKC, likely through Gq [147,161], α2-ARs activate Gi [162], SRC activates NOX2 [163], VEGFR2 activates PLC [164], PKC/MEK/ERK/EGR1 signaling activates the transcription of ACE [165,166], ${\mathrm{Ca}}^{2+}$ import through TRPV4 mediates trauma-induced endothelial barrier permeability [146], ROCK inhibits MLCP [167], DAG activates PKC [168], PKC activates RAF [169], RAS/RAF/MEK/ERK signaling contributes to endothelial permeability [170], ${\mathrm{Ca}}^{2+}$ activates PKC [171], PKC inhibits MLCP through CKI-17, increasing MLC activity [172], The ${\mathrm{Ca}}^{2+}$/Cam/MLCK/MLC pathway increases permeability [167,173], ${\mathrm{Ca}}^{2+}$ activates NOX5 contributing to ROS production [174], HIF1 increases NOX4 expression, resulting in ROS production [56], uncoupled eNOS produces superoxide [175], VEGFA activates VEGFR2 [176], the VEGFA/VEGFR2/STAT3/VEGFA feedback mechanism [177]
ECs cultivated with flow	RHOA activates ROCK, leading to endothelial barrier dysfunction [178]
Human non-ECs	Gq signaling activates PLA2 [179], α2-AR-activated SRC increases ERK activity through RAS/RAF [63], ERK activates EGR1 through ELK1, an ETS transcription factor [180]
Mice	CREB increases TH, DDH, DβH, and PNMT transcription in ECs [160], VEGFR2 activates SRC through TSAd increasing permeability [181,182], α2-ARs-induced Gi activates SRC [63,183], Angiotensin II -activated AT-1 activates PKC, likely through Gq [161], NE activates DRD1 [184], Gq signaling activates NOX2 [185], VEGFR2 activates PLC [164], Gq-stimulated PLC activates TRPV4 by consuming PIP2 [115], RHOA activates ROCK, leading to endothelial barrier dysfunction [178], α2-AR-activated SRC increases ERK activity through RAS/RAF [63], Cav imports ${\mathrm{Ca}}^{2+}$ [186], Angiotensin II activates NOX2, increasing oxidative stress [187,188], uncoupled eNOS produces superoxide [189], ${\mathrm{Ca}}^{2+}$ inhibits AC6 [190]
Rats	AT-1 activates Gq [191], α2-ARs activate Gi [192], β-AR signaling preserves endothelial barrier function [193], PKC activates RAF [194,195], COX1 and COX2 increase oxidative stress [54], ${\mathrm{Ca}}^{2+}$ inhibits AC6 [196]
Hamsters	PKA phosphorylates β-ARs to activate Gi [197]
Bovine	Gq activates PLCβ-1 [198], DAG activates PKC [168], RAS/RAF/MEK/ERK signaling contributes to endothelial permeability [170]
Human molecular evidence	Catecholamine synthesis [199], AR binding affinity [200], NE and EPI bind DRD2 and DRD4, reducing cAMP production [201], α1-ARs activate Gq [53,202], PKA-phosphorylated β-ARs activate Gi [197], ${\mathrm{Ca}}^{2+}$-induced β-ARs activate Gi [203], β-ARs activate Gs [197,203], DRD1 [204] and DRD5 [145] activate Gs, α1-AR/Gq/PKC signaling increases ROS production [51], Gq activates PLCβ-1 [205], PLCβ1 catalyzes the production of IP3 and DAG from PIP2 [206], ACE produces Angiotensin II [207], IP3 binds and opens IP3R, causing the release of ${\mathrm{Ca}}^{2+}$ from the endoplasmic reticulum [208], ${\mathrm{Ca}}^{2+}$-bound CAM activates eNOS [209], oxygen activates PHD1 leading to the degradation of HIF1A [210], HIF1 activates the expression of VEGFA [211], VEGFA activates VEGFR2 [176,212]

## Data Availability

No new data were created or analyzed in this study. Data sharing is not applicable to this article.

## Supplementary Materials

The following supporting information can be downloaded at: https://www.mdpi.com/article/10.3390/ijms27167210/s1.

## Abbreviations

The following abbreviations are used in this manuscript:

AA	arachidonic acid
AC	adenylyl cyclase
ACE	angiotensin converting enzyme
ACTH	adrenocorticotropic hormone
ADH	vasopressin
AJ	adherence junction
APC	activated protein C
AR	adrenergic receptor
CAM	calmodulin
cAMP	cyclic adenosine monophosphate
COMT	catechol-O-methyltransferase
COX	cyclooxygenase
CREB	cyclic AMP-responsive element-binding protein
CRH	corticotropin-releasing hormone
DAG	diacylglycerol
DAMP	damage associated molecular pattern
EC	endothelial cell
eNOS	endothelial nitric oxide synthase
EPCR	endothelial protein C receptor
EPI	epinephrine
ETS	E26 transformation-specific
$G_{\alpha }$	guanine nucleotide-binding protein subunit α
HIF	hypoxia-inducible factor
HSF1	heat shock transcription factor 1
HSPG	heparan sulfate proteoglycan
ICAM	intercellular adhesion molecule
MAO	L-monoamine oxidase
MLC	myosin light chain
MMP	matrix metalloproteinases
MOF	multiorgan failure
NE	norepinephrine
NOX	NADPH oxidase
PAMP	pathogen associated molecular pattern
PC	protein C
PECAM-1	platelet endothelial cell adhesion molecule-1
PIEZO	piezo-type mechanosensitive ion channel
PK	protein kinase
PLA2	phospholipase A2
RAC1	Ras-related C3 botulinum toxin substrate 1
RBC	red blood cells
RHOA	ras homolog family member A
ROS	reactive oxygen species
SHINE	shock induced endotheliopathy
SK	small conductance, calcium-activated potassium channels
SNS	sympathetic nervous system
SRC	a non-receptor tyrosine kinase
sTM	soluble thrombomodulin
TF	tissue factor
TH	tyrosine hydroxylase
Th2	T helper 2 cells
TIC	trauma-induced coagulopathy
TJ	tight junction
TM	thrombomodulin
tPA	tissue plasminogen activator
TRPA	transient receptor potential ankyrin
TRPV	transient receptor potential vanilloid
VCAM	vascular cell adhesion molecule
VE-cadherin	vascular endothelial cadherin
VEGF	vascular endothelial growth factor
WPB	Weibel-Palade bodie
ZO	zona occludens
